# Supplementary material for: Modeling glioblastoma heterogeneity as a dynamic network of cell states
Source: Mol Syst Biol. 2021 Sep 16;17(9):e10105. doi: 10.15252/msb.202010105 (PMC8444284; doi:10.15252/msb.202010105)
Supplement: Supplementary file 6 — Source Data for Figure 5 [file MSB-17-e10105-s004.zip › Figure5A_sourcedata/GSEA_3017/nosigdb_stateD.GseaPreranked.1622795010397/IVY_CT.html]

Details for gene set IVY\_CT[GSEA]

|  || Dataset | state23017 |
| Phenotype | NoPhenotypeAvailable |
| Upregulated in class | na\_neg |
| GeneSet | IVY\_CT |
| Enrichment Score (ES) | -0.54440355 |
| Normalized Enrichment Score (NES) | -2.953687 |
| Nominal p-value | 0.0 |
| FDR q-value | 0.0 |
| FWER p-Value | 0.0 |
Table: GSEA Results Summary

  

Fig 1: Enrichment plot: IVY\_CT      
 Profile of the Running ES Score & Positions of GeneSet Members on the Rank Ordered List

  

| PROBE | GENE SYMBOL | GENE\_TITLE | RANK IN GENE LIST | RANK METRIC SCORE | RUNNING ES | CORE ENRICHMENT || 1 | SOX11 |  |  | 6 | 0.578 | 0.0060 | No |
| 2 | OLIG1 |  |  | 10 | 0.517 | 0.0133 | No |
| 3 | PTTG1 |  |  | 14 | 0.490 | 0.0201 | No |
| 4 | DSEL |  |  | 16 | 0.472 | 0.0281 | No |
| 5 | MEX3A |  |  | 30 | 0.402 | 0.0251 | No |
| 6 | ID4 |  |  | 38 | 0.374 | 0.0264 | No |
| 7 | DBI |  |  | 57 | 0.339 | 0.0182 | No |
| 8 | HES6 |  |  | 60 | 0.336 | 0.0228 | No |
| 9 | OLIG2 |  |  | 98 | 0.293 | -0.0018 | No |
| 10 | BIRC5 |  |  | 130 | 0.271 | -0.0220 | No |
| 11 | LRRN1 |  |  | 151 | 0.258 | -0.0334 | No |
| 12 | SHOX2 |  |  | 308 | -0.264 | -0.1556 | No |
| 13 | MSI1 |  |  | 332 | -0.267 | -0.1693 | No |
| 14 | SPATA6 |  |  | 383 | -0.272 | -0.2049 | No |
| 15 | GATM |  |  | 384 | -0.272 | -0.1998 | No |
| 16 | UHRF1 |  |  | 438 | -0.278 | -0.2378 | No |
| 17 | LFNG |  |  | 452 | -0.280 | -0.2431 | No |
| 18 | DDR1 |  |  | 491 | -0.284 | -0.2688 | No |
| 19 | MCM10 |  |  | 515 | -0.287 | -0.2821 | No |
| 20 | CKB |  |  | 554 | -0.292 | -0.3076 | No |
| 21 | CSPG5 |  |  | 588 | -0.297 | -0.3289 | No |
| 22 | TSC22D4 |  |  | 602 | -0.301 | -0.3338 | No |
| 23 | PRC1 |  |  | 645 | -0.310 | -0.3622 | No |
| 24 | DNMT1 |  |  | 659 | -0.312 | -0.3670 | No |
| 25 | C21orf62 |  |  | 687 | -0.316 | -0.3830 | No |
| 26 | ITGB8 |  |  | 694 | -0.318 | -0.3819 | No |
| 27 | MLC1 |  |  | 701 | -0.320 | -0.3808 | No |
| 28 | KIF4A |  |  | 706 | -0.320 | -0.3780 | No |
| 29 | SDC3 |  |  | 744 | -0.329 | -0.4020 | No |
| 30 | HIST1H2AG |  |  | 786 | -0.339 | -0.4290 | No |
| 31 | CDC20 |  |  | 793 | -0.341 | -0.4275 | No |
| 32 | FANCI |  |  | 801 | -0.345 | -0.4267 | No |
| 33 | CRISPLD1 |  |  | 807 | -0.347 | -0.4242 | No |
| 34 | BLM |  |  | 812 | -0.348 | -0.4209 | No |
| 35 | CENPM |  |  | 818 | -0.349 | -0.4185 | No |
| 36 | NLGN1 |  |  | 875 | -0.364 | -0.4572 | No |
| 37 | HIST1H2AM |  |  | 918 | -0.381 | -0.4843 | No |
| 38 | LHFPL3 |  |  | 919 | -0.381 | -0.4771 | No |
| 39 | DTL |  |  | 956 | -0.397 | -0.4990 | No |
| 40 | DCLK2 |  |  | 986 | -0.408 | -0.5149 | No |
| 41 | KCNIP1 |  |  | 1018 | -0.426 | -0.5322 | No |
| 42 | TPX2 |  |  | 1034 | -0.437 | -0.5362 | Yes |
| 43 | EDNRB |  |  | 1042 | -0.440 | -0.5336 | Yes |
| 44 | WSCD1 |  |  | 1054 | -0.447 | -0.5342 | Yes |
| 45 | KLHDC8A |  |  | 1059 | -0.451 | -0.5289 | Yes |
| 46 | CDKN2C |  |  | 1067 | -0.454 | -0.5261 | Yes |
| 47 | SEMA6A |  |  | 1069 | -0.455 | -0.5184 | Yes |
| 48 | EZH2 |  |  | 1070 | -0.455 | -0.5098 | Yes |
| 49 | GRIK3 |  |  | 1078 | -0.459 | -0.5068 | Yes |
| 50 | ASCL1 |  |  | 1097 | -0.467 | -0.5127 | Yes |
| 51 | BUB1B |  |  | 1111 | -0.477 | -0.5143 | Yes |
| 52 | MEOX2 |  |  | 1118 | -0.486 | -0.5101 | Yes |
| 53 | TTK |  |  | 1119 | -0.487 | -0.5009 | Yes |
| 54 | ASF1B |  |  | 1133 | -0.498 | -0.5021 | Yes |
| 55 | TTYH1 |  |  | 1144 | -0.505 | -0.5008 | Yes |
| 56 | CKAP2L |  |  | 1147 | -0.508 | -0.4928 | Yes |
| 57 | FAM111B |  |  | 1151 | -0.514 | -0.4856 | Yes |
| 58 | NUSAP1 |  |  | 1154 | -0.515 | -0.4776 | Yes |
| 59 | KIF14 |  |  | 1168 | -0.535 | -0.4781 | Yes |
| 60 | KIF2C |  |  | 1183 | -0.558 | -0.4790 | Yes |
| 61 | CDCA3 |  |  | 1187 | -0.563 | -0.4708 | Yes |
| 62 | ASPM |  |  | 1189 | -0.567 | -0.4610 | Yes |
| 63 | NUF2 |  |  | 1194 | -0.574 | -0.4534 | Yes |
| 64 | DHFR |  |  | 1197 | -0.576 | -0.4442 | Yes |
| 65 | GTSE1 |  |  | 1210 | -0.602 | -0.4427 | Yes |
| 66 | CLSPN |  |  | 1212 | -0.605 | -0.4321 | Yes |
| 67 | KIF15 |  |  | 1213 | -0.610 | -0.4206 | Yes |
| 68 | CENPK |  |  | 1220 | -0.619 | -0.4139 | Yes |
| 69 | SMC4 |  |  | 1224 | -0.638 | -0.4043 | Yes |
| 70 | MYBL2 |  |  | 1225 | -0.639 | -0.3923 | Yes |
| 71 | RAMP1 |  |  | 1230 | -0.648 | -0.3833 | Yes |
| 72 | CDCA8 |  |  | 1239 | -0.669 | -0.3773 | Yes |
| 73 | MELK |  |  | 1251 | -0.701 | -0.3730 | Yes |
| 74 | LRRC17 |  |  | 1253 | -0.711 | -0.3605 | Yes |
| 75 | CDCA2 |  |  | 1258 | -0.727 | -0.3500 | Yes |
| 76 | HMGB2 |  |  | 1263 | -0.736 | -0.3394 | Yes |
| 77 | RAD51AP1 |  |  | 1264 | -0.741 | -0.3255 | Yes |
| 78 | TACC3 |  |  | 1268 | -0.751 | -0.3138 | Yes |
| 79 | AURKA |  |  | 1271 | -0.763 | -0.3011 | Yes |
| 80 | CCNE2 |  |  | 1273 | -0.771 | -0.2874 | Yes |
| 81 | ID3 |  |  | 1276 | -0.785 | -0.2742 | Yes |
| 82 | NDC80 |  |  | 1278 | -0.810 | -0.2598 | Yes |
| 83 | ESCO2 |  |  | 1283 | -0.846 | -0.2472 | Yes |
| 84 | LRRN3 |  |  | 1293 | -0.932 | -0.2369 | Yes |
| 85 | RRM2 |  |  | 1295 | -0.933 | -0.2202 | Yes |
| 86 | FBXO5 |  |  | 1301 | -0.992 | -0.2056 | Yes |
| 87 | NCAPG |  |  | 1303 | -1.015 | -0.1873 | Yes |
| 88 | LPL |  |  | 1304 | -1.038 | -0.1678 | Yes |
| 89 | HIST1H2AH |  |  | 1305 | -1.040 | -0.1482 | Yes |
| 90 | CDK1 |  |  | 1311 | -1.433 | -0.1253 | Yes |
| 91 | UBE2C |  |  | 1312 | -1.466 | -0.0977 | Yes |
| 92 | TOP2A |  |  | 1313 | -1.528 | -0.0690 | Yes |
| 93 | HIST1H1B |  |  | 1318 | -1.922 | -0.0360 | Yes |
| 94 | HIST1H1D |  |  | 1319 | -1.958 | 0.0008 | Yes |
Table: GSEA details [plain text format]

  

Fig 2: IVY\_CT: Random ES distribution      
 Gene set null distribution of ES for **IVY\_CT**
